# 

**Published:** 1782

**Authors:** 


### P R O M O T E D.

*June* 24. Messieurs John Theodore van der Kemp, Harper Hall, Andrew Sayer, Edward Hart, William Gourlay, William Corp, John R. Fenwick, George Paton, and Jonathan Stokes, to be Doctors of Physic in the University of Edinburgh.—29. Charles Grove and John Caulet of St. John's College, and Henry Topping of King's College, Bachelors of Physic, to be Doctors of Physic in the University of Cambridge. John Billam of Trinity College, and Thomas Norford of Caius College, Masters of Arts, to be Bachelors of Physic in the same University.

*July* 6. John Matthews of Merton College, John Bree of University College, and John Littlehales of Pembroke College, Bachelors of Physic, to be Doctors of Physic in the University of Oxford.—29. Robert Scott, M. D. physician, and and (*Auguſt* 9) Henry Jebb, Eſq; ſurgeon at Dublin, to the rank of Knighthood by the Lord Lieutenant of Ireland.

*Auguſt* 26. Mr. Thomas White to be ſurgeon to the Military Aſſociation at Mancheſter.—28. John Parſons, M. D. to be phyſician to the Finſbury Diſpenſary in London.

*September* 7. Mr. Thomas Cawley, aſſiſtant ſurgeon, to be chief ſurgeon to the forces in Jamaica, in the room of Mr. Edward Horler.—9. Meſſieurs Richard Vaughan, George Danſell, Philip Holland, James Hutchinſon, James Hare, Andrew Marſhall, Daniel Bryan, Samuel de Butts, Thomas Evory, Henry Garde, and James Forſythe, to be Doctors of Phyſic in the Univerſity of Edinburgh.

---

### D I E D.

1781. *December.* At Luneville in Lorraine, M. Pierre, M. D. correſponding member of the Royal College of Phyſicians at Nancy.

1782. *January.* At Bourbonne, M. Tailler, M.D. correſponding member of the ſame College, and author of ſeveral diſſertations.

*June.* Mr. George Clarke, apothecary in St. Alban's-ſtreet, London.—21. At Chowbent, in Lanca-

Lancashire, Mr. Richard Guest, surgeon and apothecary.—23. At Durham, aged 33 years, Thomas Blackburne, M. D. F. R. S. member of the Medical Societies of London and Edinburgh. He was a son of the Rev. Francis Blackburne, the present worthy and learned Archdeacon of Cleveland; and was born at Richmond in Yorkshire, where his father is Rector. He received the rudiments of his education in that town, and went from thence to the Charter-House, on the recommendation of the late Marquis of Rockingham. After two years spent in that excellent seminary, he was admitted a scholar of St. Peter's College, Cambridge; and being of standing to take the degree of B. A. he underwent a public examination in the Senate-House with high approbation: but finding he could not in conscience subscribe the Thirty-nine Articles, he quitted the University without a degree, and turning his studies to Physic, pursued them at London and Edinburgh, at which University he took his Doctor's degree, and about five years ago settled at Durham, where his memory will be long respected on account of his professional abilities, his learning in other branches of science, his urbanity of manners in general, and his benevolence to the poor and distressed who fell within his notice. He was the author of an ingenious inaugural diſſertation "*de Medici inſtitutis*," written in very claſſical Latin, and printed at Edinburgh in 1775; and of an account of four caſes of Tænia ſucceſsfully treated, in a letter to Dr. Simmons, and publiſhed by the latter in an appendix to the ſecond edition of his work on the Tænia. This Journal likewiſe, to which he was an early contributor, is indebted to him for two intereſting papers, which alone would be ſufficient to make us regret the death of ſo valuable and promiſing a member of the medical profeſſion.—24. At Kingſton in Jamaica, Dr. Beaumont Topott.

*July* 1. Dr. Joſiah Gibbons, ſurgeon of his majeſty's ſhip the Glorieux, drowned in a ſtorm by the overſetting of a wherry between Kingſton and Port Royal in Jamaica. He was a native of Georgia in America, and ſtudied phyſic at Edinburgh, where he graduated in September 1776. His theſis on that occaſion was intituled, *De quibuſdam puerperarum Morbis.*—24. At Merry-field near Plymouth, Mr. Henry Thompſon, a ſurgeon of great ability and eminence, and author of ſeveral very uſeful and ingenious papers publiſhed in different volumes of the Medical Obſervations and Inquiries. He reſigned his poſt of of ſurgeon to the London Hoſpital, and retired from practice but a little time before his death. The writer of this article remembers being preſent at the London Hoſpital, in the year 1768, at the amputation of a man's arm at the joint of the ſhoulder, by Mr. Thompſon; an operation, in which he diſplayed all that coolneſs and dexterity that characterize the true ſurgeon.

*Auguſt* 3. At Bradford in Yorkſhire, Edmund Simpſon, M. D. He graduated at Edinburgh in September 1779; on which occaſion he was the author of a theſis *de Oculo humano.* —13. At Naples, Alexander Monro Drummond, M. D. a phyſician of great learning and abilities. He was the ſon of an eminent bookſeller at Edinburgh, and in 1760 was admitted of the Medical Society in that city. From that time till 1771, when he graduated, he officiated as phyſicians clerk at the Infirmary. During the whole of this period he was remarkable for his unremitting attention to his profeſſional purſuits. In 1771 he quitted Scotland, and viſited the continent of Europe, where his talents and addreſs procured him the friendſhip of ſome of the moſt diſtinguiſhed Britiſh travellers. He accompanied Lord Algernon Percy into Greece, and returned from thence to Naples, where he remained till his death. As a proof of the eſtimation in which his profeſſional abilities were held by his countrymen, it will be ſufficient to mention, that in May 1773, when he had been near two years abroad, the magiſtrates of Edinburgh elected him to the medical profeſſorſhip vacant by the death of Dr. Gregory; and that they did not proceed to a freſh election till June 1776, and even then not without regretting that Dr. Drummond abſolutely declined accepting their offer. At Naples he was highly eſteemed. There are anecdotes related of him, which prove that he was as diſintereſted as Jean Jaques Rouſſeau, and in many reſpects as eccentric. He was often invited to return to England, and Lord Winchelſea had once ſeemingly prevailed on him to accompany him to London; but when the time for departure arrived, the doctor contrived to excuſe himſelf from being of the party. A little before we ſaw his death announced in the Newſpapers, he was ſaid to be employed in writing an account of his travels. He was author of an inaugural theſis, entitled, *Commentarius de febribus arcendis diſcutiendiſque.*—18. At Hathem near Loughborough in Leiceſterſhire, aged 74 years, Mr. Bowley,

Bowley, ſurgeon and apothecary. — 23. At Leeds in Yorkſhire, aged 58, William Hird, M. D. a quaker phyſician of conſiderable eminence as a practitioner. He graduated at Edinburgh in 1751, and on that occaſion defended a theſis *de Lactis natura & uſu.* He was a nephew of the late Dr. John Fothergill, who, it is ſaid, intended to have reliquiſhed practice in his favour. Immediately after the death of that celebrated phyſician Dr. Hird quitted Leeds in order to ſettle himſelf in London, where he placed himſelf in the houſe of his deceaſed relation in Harpur-ſtreet. Here he publiſhed "An affectionate tribute to the memory of the "late Dr. John Fothergill," which is written in a ſenſible and pleaſing manner. He remained, however, only a very few weeks in his new ſituation. He foreſaw, that the forming new connexions would be a work of time, and that the progreſs of a phyſician in this metropolis is ſlow, and but too often dependent on accidental circumſtances. Theſe conſiderations induced him to return to Leeds, where his profeſſional talents were known and reſpected. — 29. Mr. James Bertram, ſurgeon of his majeſty's ſhip the Royal George, and one of the unforunfortunate perfons who were on board that veffel when it funk at Portfmouth.

*September.* At Holloway Down near Stratford, John Harris, M. D.—2. In Bifhopfgate-ftreet, London, Mr. Armftrong, furgeon.—6. At Nottingham, after a lingering illnefs, Mr. James Bigfby, furgeon and apothecary.—8. Mr. Cecil, apothecary in Southwark.—12. At Malton in Yorkfhire, aged 48 years, Mr. John Temple, furgeon and apothecary.—22. At Windfor, John Thackeray, M. B.

---

### SECTION IV.

### QUARTERLY CATALOGUE.

1. P. *M. Augufti Brouffonet,* Medicinæ Doctoris, Societat. Reg. Londinenfis et Monfpelienfis Socii, Ichthyologia fiftens Pifcium defcriptiones et Icones. Large 4to. *Elmfley*, London, 1782. 10s. 6d.

The fubject of this work does not properly come within the plan of our Journal, but we have thought it right to announce it as being a performance of great merit in Natural Hiftory, a fcience in the progrefs of which medical readers readers cannot but feel themſelves particularly intereſted.

What is now publiſhed is only the firſt Decade, which is to be followed by others till the work is completed. It contains deſcriptions and engravings of ten fiſhes (chiefly from the South Sea) from ſpecimens in the poſſeſſion of Sir Joſeph Banks, Bart to whom the work is inſcribed. Half a ſheet of letter preſs and a ſeparate plate are allotted to each fiſh. The deſcriptions appear to be extremely exact, and by means of the late Dr. Solander's MSS. notes the author has been enabled to mark the genuine colours of the different ſpecimens. The engravings are very elegant, and without doubt very accurate, as they were executed under the immediate inſpection of the ingenious author.

2. New thoughts on Medical Electricity; or an attempt to diſcover the real uſes of electricity in medicine; including a very remarkable caſe. In two letters to a friend. 8vo. *Cumberledge*, London, 1782. 1s.

3. Obſervations on the Sulphur Water at Croft near Darlington. By *Robert Willan*, M. D. 8vo. *Johnſon*, London, 1782. 1s. 6d.

This little work is a uſeful addition to the hiſtory of our medicinal waters. According to the the accurate analyfis here given of the Croft water, it contains fulphur, a fmall portion of iron, a confiderable quantity of Epfom falt, a little common falt, calcareous earth, and fixed air.

4. An inquiry into the ftate of Medicine on the true principles of inductive Philofophy; with an appendix containing practical cafes and obfervations. By *Robert Jones*, M. D. 8vo, *Longman*, London, 1782. 6s.

5. An account of fome experiments on mercury, filver, and gold, made at Guildford in May 1782 in the laboratory of *James Price*, M. D. F. R. S. To which is prefixed an abridgment of Boyle's account of a degradation of gold. 4to. Oxford 1782. 28 pages. 1s.

Thefe experiments relate to fome very extraordinary changes in mercury and filver by means of a red and a white powder, neither of which the author has thought fit to defcribe. By adding only half a grain of the red powder to half an ounce of mercury in a flux of borax and charcoal, the mercury was prevented from boiling although in a red heat; and in another experiment, when actually boiling and evaporating, it was almoft inftantaneoufly fixed by the addition of the fame fubftance. Ten grains of pure pure gold were found in the ſcoriæ. Half a grain of the white powder employed in the ſame manner produced fifteen grains of ſilver. Theſe experiments, we are told, were performed in the preſence of Lords Onſlow, King, and Palmerſtone; Sir R. Barker, Sir P. N. Clarke, Dr. Spence, Mr. Godſchall, and other perſons of credit.

In the introduction to the work we meet with the following paſſage: "The whole of the ma-"terials producing the extraordinary change in "the metal employed, was expended in per-"forming the proceſſes which are now to be "related; nor can the author furniſh himſelf "with a ſecond portion, but by a proceſs "equally tedious and operoſe, whoſe effects he "has recently experienced to be injurious to his "health, and of which he muſt therefore avoid "the repetition."

6. Lettre ſur les experiences des frictions glaciales, pour la guerifon de la peſte et autres maladies putrides. *i. e.* A letter concerning experiments on the efficacy of icy frictions in the cure of the Plague and other putrid diſorders. By *D. Samoïlowitz*, M. D. Aſſeſſor of the Colleges of her Imperial Majeſty of all the Ruſſias, Surgeon major to the Senate of Moſcow, and Member of the Commiſſion for providing againſt the Plague. 8vo. Paris, 1781. 54 pages.

The plague, that made ſuch havoc in the Ruſſian dominions, and particularly at Moſcow in 1771, we are told, gave occaſion to the publication of this letter. The author addreſſes it to the phyſicians of Europe, in order to announce a work which he is about to publiſh, and in which he promiſes to give a minute account of the external uſe of ice in the cure of the plague. An extract from the work in queſtion is annexed to his epiſtle, containing three caſes in which the remedy proved efficacious. One of the patients was attacked with a peſtilential bubo, another with a carbuncle on the breaſt, and the third with petechiæ and carbuncles.

The idea of applying ice in the plague took its riſe, we are told, from the cuſtom which has long been ſucceſsfully followed in Ruſſia, of rubbing frozen limbs with ſnow. In honour of the preſent Empreſs of Ruſſia, by whoſe order, it ſeems, this remedy was firſt introduced as a cure for the plague, our author names it *Antipeſtilentiale Catharinæ*. We muſt leave it to time and farther experience to determine the merits of this new remedy. In the mean mean time we may obſerve, that Thucydides in his account of the plague at Athens, particularly obſerves that neither the internal nor external uſe of very cold water prevented the ſick from dying; and that Vinarius and Guy de Chauliac, two writers of the 14th century, who had repeated opportunities of treating the plague, obſerve, that the external application of cold prevented the eruption of exanthemata and buboes, and by this means always proved fatal.

7. Torberni Bergman, Chemiæ Prof. Upſ. et Equitis aurati Regii ordinis de Waſa, *Opuſcula Phyſica et Chemica, pleraque ſeorſim antea edita, jam ab auctore collecta, reviſa, et aucta.* Vol. II. *cum tabulis æneis.* 8vo. Upſal. 1780. p. 510.

Of the firſt volume of this excellent collection of philoſophical and chemical eſſays we gave an account in our Journals for January and February 1781. This ſecond volume contains the following diſſertations: 1. *De formis chryſtallorum, præſertim e ſpatho ortis*; 2. *De Terra Silicea*; 3. *De lapide hydrophano*; 4. *De Terra Gemmarum*; 5. *De Terra Turmalini*; 6. *De Calce auri fulminante*; 7. *De Platina*; 8. *De mineris ferri albis*; 9. *De Nicolo*; 10. *De Arſenico*; 11.

 *De*

*De mineris Zinci*; 12. *De præcipitatis metallicis*; 13. *De minerarum Docimasia humida*; 14. *De Tubo Ferruminatorio, ejusdemque usu in explorandis corporibus, præsertim mineralibus.*

8. Gerichlich-Medinische beobachtugen, *i. e.* Cases relative to medical jurisprudence. By *M. Metzer*, M. D. &c. 8vo. Koningsberg, 1780. 192 pages.

9. Einleitungin die pharmacie, *i e.* An Introduction to pharmacy. By *John Frederick Gmelin*, M. D. Professor of Chemistry in the University of Gottingen, &c. 8vo. Nuremberg, 1781. 392 pages.

10. *Nicol. Rigler*, Physici Ducatus Bielicensis in Silesiâ Austriacâ, Constitutio Epidemica annorum 1775, 1776, 1778, 1779, adjectis nonnullis selectioribus casibus practicis. 8vo. Breslaw, 1780. p. 158.

11. Dissertatio Inauguralis de sanguinis missione in febribus putridis. Auctore *Wernero Ernesto Rudolphi.* 4to. Gottingæ, 1780. p. 32.

12. Dissertatio Inauguralis de pleuritide. Auctore *Gulielmo Broc.* 4to. Nancy, 1781.

13. Catalogue Systematique des arbres & arbustes etrangers, la plupart de l'Amerique Septen-

Septentrionale, cultivés dans le jardin Americain a la terre de Madame la Comtesse de Hohenheim, & qui passent l'hiver en plein champ, *i. e.* A systematic Catalogue of exotic trees and shrubs, the greater part of them from North America, cultivated in the American garden belonging to the Countess of Hohenheim, and which pass the winter in the open air. 16mo. Stutgard, 1780. 255 pages.

This work contains a list of 850 trees and shrubs, arranged according to the Linnæan system. The names are in Latin, French, and German.

14. Observationum obstetriciarum de partu clunibus præviis peracto decas. Auctore *Georgio Gulielmo Spangerberg*. 4to. Gottingen, 1781.

15. Description, usage et avantages de la machine pour reduire les fractures des jambes, inventée par Dom. Alb. Pieropan de Vicence, *i.e.* Description, mode of application, and advantages of the machine invented for the reduction of fractured legs, by Father Alb. Pieropan of Vicenza. By *M. Mongez* jun. Member of several Academies, and Author of the Journal de Physique. 8vo. Paris, 1782. 22 pages, with a plate.

This

This machine has been approved by the College of Health at Venice. Medals have been ſtruck in honour of the inventor, and it is uſed in all the hoſpitals in the Venetian territories. It conſiſts of two pieces of braſs reaching, on each ſide of the leg, from a band and cuſhion which ſurround the thigh a little above the knee, and terminating in a ſhoe. The braſs plates are furniſhed with a ſcrew, by means of which they may be lengthened or ſhortened.

16. Pharmacopeia Pauperum, in uſum Inſtituti clinici Hamburgenſis, edita a ſocietate medica. 8vo. Hamburgh, 1781. p. 71.

This is a judicious compilation. The Inſtitution conſiſts of ſeven phyſicians and five ſurgeons, who viſit the patients, and of five apothecaries who prepare the medicines gratis.

17. Medicina Clinica, auctore *Chriſtian Gottlieb Selle*, M. D. 8vo. Berlin, 1781. p. 566.

18. Acta Societatis Jablonovianæ varii argumenti ab anno 1775 ad annum 1779. Tom. v. 4to. Leipſic, 1781. p. 296, with two copperplates.

The only medical papers in this collection are, 1. *De lue bovina Sententia*, auctore C. G. Barthio, M. D. 2. Diſſertatio de lue bovina, auctore A. M. Birkholz, M. D. 3. Diſſertatio de influxu fluxu lucis in vegetationem plantarum, auctore G. M. Ludwig.

19. Auſerleſene Vollſtoendige abhandlungen von der Colik von Poitou; *i. e.* Select Diſſertations on the Colic of Poitou, tranſlated into German from the Latin of de Haen, Graſhuys, Tronchin, and Strack. By *C. F. Schweder.* 8vo. Copenhagen, 1781. 378 pages.

20. Diſſertatio Inauguralis de Thermis Aquis Granenſibus earumque uſu ſalubri vel noxio. Auctore *Johanne le Soinne.* 4to. Duiſbourg, 1781.

21. Notions Elementaires de Botanique, avec l'explication d'une carte compoſeé pour ſervir aux cours publics de l'Academie de Dijon; *i. e.* Elements of Botany, with the explanation of a map compoſed for the public lectures of the Academy of Dijon. 8vo. Dijon, 1781. 398 pages.

This work, which is very judiciouſly executed, is written by M. Durande, Phyſician and Profeſſor of Botany at Dijon. The map exhibits the different ſyſtems, and is evidently the reſult of much labour.

22. Tratado teorico-practico de Materia Medica, interna y externa, que explica los medicamentos naturales o ſimples, aſi como las præparaciones chimicas las mas uſuales, ſus doſis, ſu modo de obrar, obrar, los casos donde convienen, y sus formulas, con un supplemento a lo ultimo ; *i. e.* A theoretico-practical Treatise on the Materia Medica both internal and external, with an account of natural and simple remedies, and of the most usual chemical preparations, their doses, mode of operating, the cases in which they are indicated, and their formulæ, with a supplement to the latter. By Don *Juan Rancé*, M. D. 4to. Barcelona. 3 vols.

23. Dissertacion sobre el Sen de Espana, pruebase come especificamente no es distinto del Alexandrino ü Oriental; y explicanse sus virtudes en la Medicina, su cultivo, &c. a que se annade la lamina de la planta; *i. e.* A Dissertation on the Sena of Spain, proving it to be not specifically different from the Alexandrian or Oriental Sena; and explaining its medicinal virtues, the mode of cultivating it, &c. to which is annexed an engraving of the plant. By Don *Salvador Soliva*, M. D. 8vo. Madrid.

